# Supplementary material for: Lung microenvironments harbor Mycobacterium tuberculosis phenotypes with distinct treatment responses
Source: Antimicrob Agents Chemother. 2023 Aug 11;67(9):e00284-23. doi: 10.1128/aac.00284-23 (PMC10508168; doi:10.1128/aac.00284-23)
Supplement: Tables S1 to S5 — Supplemental results. [file aac.00284-23-s0001.docx]

**SUPPLEMENTAL INFORMATION**

**Title:** Lung microenvironments harbor *Mycobacterium tuberculosis* phenotypes with distinct treatment responses

**Authors:**

Nicholas D. Walter^1,2,3^, Jackie P. Ernest^4^, Christian Dide-Agossou^1,2^, Allison A. Bauman^5^, Michelle E. Ramey^5^, Karen Rossmassler^1,2^, Lisa M. Massoudi^5^, Samantha Pauly^1,2^, Reem AlMubarak^1,2^, Martin I. Voskuil^3,6^, Firat Kaya^7^, Jansy P. Sarathy^7^, Matthew D. Zimmerman^7^, Véronique Dartois^7^, Brendan K. Podell^3,5^, Radojka M. Savic^3,4^, and Gregory T. Robertson^3,5†^

**Affiliation:**

1. Rocky Mountain Regional VA Medical Center, Aurora, CO, USA
2. Division of Pulmonary Sciences and Critical Care Medicine, University of Colorado Anschutz Medical Campus, Aurora, CO, USA
3. Consortium for Applied Microbial Metrics, Aurora, CO, USA
4. Department of Bioengineering and Therapeutic Sciences, University of California San Francisco, San Francisco, CA, USA
5. Mycobacteria Research Laboratories, Department of Microbiology, Immunology and Pathology, Colorado State University, Fort Collins, Colorado, United States of America
6. Department of Immunology and Microbiology, University of Colorado Anschutz Medical Campus, Aurora, CO, USA
7. Center for Discovery and Innovation, Nutley, New Jersey, USA

† Corresponding author

Contents

[1. Supplemental Results 3](#_Toc125382915)

[1.1. Supplemental Table 1 3](#_Toc125382916)

[1.2. Supplemental Table 2 4](#_Toc125382917)

[1.3. Supplemental Table 3 5](#_Toc125382918)

[1.4. Supplemental Table 4 6](#_Toc125382919)

[1.5. Supplemental Table 5 7](#_Toc125382920)

# **Supplemental Results**

# **Supplemental Table 1**

**Supplemental Table 1.** Pairwise comparisons of CFU and RS Ratio across microenvironments in C3HeB/FeJ mice prior to drug treatment. The mean difference between CFU or mean fold-change between RS Ratio in the comparator microenvironment versus the reference microenvironment is shown with associated *P-*value. A positive difference indicates that CFU or the RS Ratio was higher in the comparator than in the reference. A fold-change <1 indicates that the RS Ratio was lower in the comparator than in the reference. *P-*values <0.05 are highlighted in bold.

| **Comparator** | **Reference** | **CFU** | | **RS ratio** | |
| --- | --- | --- | --- | --- | --- |
|  |  | **Log_10_ difference** | ***P-*value** | **Fold-change** | ***P-*value** |
| Caseum | Spleen | 3.2 | **<0.00001** | -3.7 | **<0.00001** |
| Caseum | Remaining Lung | 2.4 | **<0.00001** | -6.4 | **<0.00001** |
| Caseum | Airway | NA | NA | -14.5 | **<0.00001** |
| Remaining Lung | Spleen | 0.8 | **0.02** | 1.7 | **0.01** |
| Remaining Lung | Airway | 0.6 | NA | -2.2 | **0.0003** |
| Spleen | Airway | NA | NA | -3.8 | **<0.00001** |
|  |  |  |  |  |  |

# **Supplemental Table 2**

**Supplemental Table 2.** Pairwise comparisons of CFU and RS Ratio for individual drugs within specific microenvironments. The mean difference between CFU or mean fold-change between RS Ratio for each drug versus the reference is shown with associated *P-*value. A positive difference indicates that CFU or the RS Ratio was higher for the comparator drug than in the reference. A fold-change <1 indicates that the RS Ratio was lower in the comparator than in the reference. *P-*values <0.05 are shown in bold.

| **Comparator** | **Reference** | **CFU** | | **RS Ratio** | |
| --- | --- | --- | --- | --- | --- |
|  |  | **Mean log_10_ difference** | ***P-*value** | **Mean**  **fold-change** | ***P-*value** |
| **Caseum** | | | | | |
| INH | CONTROL | -1.00 | **0.02** | 1.19 | 1.0 |
| RIF10 | CONTROL | -1.00 | **0.06** | -2.61 | **0.04** |
| RIF30 | CONTROL | -1.48 | **0.005** | -4.05 | **0.003** |
| BDQ | CONTROL | -1.45 | **0.0003** | -1.00 | 1.0 |
| RIF10 | INH | 0.00 | 1.0 | -3.09 | **0.005** |
| RIF30 | INH | -0.48 | 0.7 | -4.80 | **0.0004** |
| BDQ | INH | -0.45 | 0.4 | -1.19 | 1.0 |
| RIF30 | RIF10 | -0.48 | 0.7 | -1.55 | 0.7 |
| BDQ | RIF10 | -0.45 | 0.6 | 2.61 | **0.02** |
| BDQ | RIF30 | 0.03 | 1.0 | 4.05 | **0.001** |
| **Spleen** | | | | | |
| INH | CONTROL | -1.85 | **<0.00001** | -5.63 | **<0.00001** |
| RIF10 | CONTROL | -2.03 | **<0.00001** | -9.18 | **<0.00001** |
| RIF30 | CONTROL | -2.87 | **<0.00001** | -20.43 | **<0.00001** |
| BDQ | CONTROL | -3.39 | **<0.00001** | -33.53 | **<0.00001** |
| RIF10 | INH | -0.17 | 0.8 | -1.63 | 0.1 |
| RIF30 | INH | -1.02 | **<0.00001** | -3.63 | **<0.00001** |
| BDQ | INH | -1.54 | **<0.00001** | -5.95 | **<0.00001** |
| RIF30 | RIF10 | -0.84 | **0.00005** | -2.22 | **0.002** |
| BDQ | RIF10 | -1.37 | **<0.00001** | -3.65 | **<0.00001** |
| BDQ | RIF30 | -0.52 | 0.2 | -1.64 | 0.1 |
| **Remaining Lung** | | | | | |
| INH | CONTROL | -1.50 | **0.0002** | -3.48 | **<0.00001** |
| RIF10 | CONTROL | -2.37 | **<0.00001** | -15.46 | **<0.00001** |
| RIF30 | CONTROL | -3.60 | **<0.00001** | -32.63 | **<0.00001** |
| BDQ | CONTROL | -3.83 | **<0.00001** | -22.13 | **<0.00001** |
| RIF10 | INH | -0.87 | 0.05 | -4.44 | **<0.00001** |
| RIF30 | INH | -2.10 | **<0.00001** | -9.37 | **<0.00001** |
| BDQ | INH | -2.33 | **<0.00001** | -6.35 | **<0.00001** |
| RIF30 | RIF10 | -1.23 | **0.002** | -2.11 | **0.0002** |
| BDQ | RIF10 | -1.46 | **0.0002** | -1.43 | 0.2 |
| BDQ | RIF30 | -0.22 | 1.0 | 1.47 | 0.1 |
| **Airway** | | | | | |
| INH | CONTROL | -2.01 | **0.003** | -3.04 | **0.01** |
| RIF10 | CONTROL | -2.16 | **0.001** | -7.70 | **<0.00001** |
| RIF30 | CONTROL | -2.40 | **0.01** | -41.65 | **<0.00001** |
| BDQ | CONTROL | -2.38 | **0.0005** | -10.57 | **<0.00001** |
| RIF10 | INH | -0.15 | 1.0 | -2.53 | 0.09 |
| RIF30 | INH | -0.38 | 1.0 | -13.69 | **<0.00001** |
| BDQ | INH | -0.37 | 1.0 | -3.47 | **0.01** |
| RIF30 | RIF10 | -0.24 | 1.0 | -5.41 | **0.004** |
| BDQ | RIF10 | -0.22 | 1.0 | -1.37 | 0.9 |
| BDQ | RIF30 | 0.02 | 1.0 | 3.94 | **0.03** |

# **Supplemental Table 3**

**Supplemental Table 3.** Pairwise comparisons of reduction in CFU and RS Ratio from control across microenvironments for individual drugs. The mean difference between CFU or mean fold-change between RS Ratio in the comparator microenvironment versus the reference microenvironment is shown with associated *P-*value. A positive difference indicates that CFU or the RS Ratio was reduced to a greater degree in the comparator than in the reference. A fold-change <1 indicates that the RS Ratio was lower in the comparator than in the reference. *P-*values <0.05 are shown in bold.

| **Comparator** | **Reference** | **CFU** | | **RS Ratio** | |
| --- | --- | --- | --- | --- | --- |
|  |  | **Mean log_10_ difference** | ***P-*value** | **Mean**  **fold-change** | ***P-*value** |
| **INH** | | | | | |
| Caseum | Spleen | -0.87 | 0.3 | -5.19 | **0.0008** |
| Caseum | Remaining lung | -0.87 | 0.3 | -2.57 | **0.006** |
| Caseum | Airway | -1.40 | **0.04** | -2.41 | **0.01** |
| Remaining lung | Spleen | 0.00 | 1.0 | -2.62 | 0.2 |
| Remaining lung | Airway | -0.52 | 0.6 | 0.17 | 1.0 |
| Spleen | Airway | -0.53 | 0.6 | 2.79 | 0.2 |
| **RIF10** | | | | | |
| Caseum | Spleen | -1.05 | 0.1 | -7.06 | **0.00007** |
| Caseum | Remaining lung | -1.75 | **0.002** | -12.72 | **<0.00001** |
| Caseum | Airway | -1.55 | **0.009** | -5.55 | **0.001** |
| Remaining lung | Spleen | 0.70 | 0.1 | 5.66 | 0.08 |
| Remaining lung | Airway | 0.20 | 0.9 | 7.18 | **0.02** |
| Spleen | Airway | -0.50 | 0.4 | 1.51 | 0.8 |
| **RIF30** | | | | | |
| Caseum | Spleen | -1.41 | **0.04** | -17.71 | **0.0004** |
| Caseum | Remaining lung | -2.50 | **0.0001** | -28.52 | **0.00008** |
| Caseum | Airway | -1.30 | 0.1 | -41.51 | **0.006** |
| Remaining lung | Spleen | 1.09 | **0.01** | 10.81 | 0.3 |
| Remaining lung | Airway | 1.20 | **0.04** | -12.99 | 0.7 |
| Spleen | Airway | 0.11 | 1.0 | -23.80 | 0.2 |
| **BDQ** | | | | | |
| Caseum | Spleen | -1.96 | **0.002** | -35.52 | **0.00001** |
| Caseum | Remaining lung | -2.75 | **<0.00001** | -21.34 | **0.00002** |
| Caseum | Airway | -1.32 | **0.004** | -10.57 | **0.0008** |
| Remaining lung | Spleen | 0.79 | 0.4 | -14.18 | 0.2 |
| Remaining lung | Airway | 1.44 | **0.0008** | 10.77 | 0.1 |
| Spleen | Airway | 0.65 | 0.6 | 24.95 | **0.004** |

# **Supplemental Table 4**

**Supplemental Table 4.** *P-*values from pairwise comparisons of the absolute RS ratio value in key microenvironments following treatment.

| **Comparator** | **Reference** | ***P-*value** |
| --- | --- | --- |
| **INH** | | |
| Caseum | Spleen | 0.4 |
| Caseum | Remaining lung | 0.5 |
| Caseum | Airway | **0.006** |
| Remaining lung | Spleen | **0.02** |
| Remaining lung | Airway | **0.04** |
| Spleen | Airway | **0.0009** |
| **RIF 10** | | |
| Caseum | Spleen | 1.0 |
| Caseum | Remaining lung | 1.0 |
| Caseum | Airway | **0.00003** |
| Remaining lung | Spleen | 1.0 |
| Remaining lung | Airway | **0.00003** |
| Spleen | Airway | **0.00002** |
| **RIF 30** | | |
| Caseum | Spleen | 0.9 |
| Caseum | Remaining lung | 0.9 |
| Caseum | Airway | 0.8 |
| Remaining lung | Spleen | 1.0 |
| Remaining lung | Airway | 0.4 |
| Spleen | Airway | 0.3 |
| **BDQ** | | |
| Caseum | Spleen | **0.0004** |
| Caseum | Remaining lung | **0.008** |
| Caseum | Airway | 0.7 |
| Remaining lung | Spleen | **0.009** |
| Remaining lung | Airway | **0.004** |
| Spleen | Airway | **0.0006** |

# **Supplemental Table 5**

**Supplemental Table 5.** Broth microdilution MIC assays conducted with *Mycobacterium tuberculosis* Erdman in 7H9 medium media supplemented with 0.2% [v:v] glycerol and 10% [v:v] ADC, with 0.05% [v:v] Tween-80 (7H9 media) plus or minus 4% [w:v] human serum albumin after 8 days incubation at 37°C. Antimicrobials tested and final test concentrations in mg/L are listed. MIC values are called as the first consecutive well showing greater than or equal to 80% growth inhibition (GI80) of the average OD at 600 nm of the no drug DMSO only control wells.

| **compound** | **MIC for Mtb Erdman in mg/L in 7H9-media with:** | |
| --- | --- | --- |
|  | **no supplement** | **4% human serum albumin** |
| rifampin | 0.008 | 0.03 |
| isoniazid | 0.03 | 0.06 |
| bedaquiline | 0.125 | 0.25 |
